# Case of Utero-Vesical Fistula—Novel Means of Relief

**Published:** 1847-11

**Authors:** 


*Case of Utero-Vesical Fistula—Novel Means of Relief.*—Mr. Isaac Harrinson brought before the Reading Pathological Society the case of a woman, aged 41 years, who, thirteen days after a protracted labour, (which had to be completed by instrumental aid,) begun to suffer from a flow of urine from the vagina, not a drop passing through its natural outlet. This state had continued, to the patient's great torment, for five years previous to her coming under Mr. Harrinson's care. After repeated examinations with the speculum, the only exact information the author could obtain was, that the urine issued from the os uteri; how it came there he was unable to determine, and had almost given it up in despair, when, exploring with a small catheter in the bladder, with the concavity downwards and a finger in the os uteri, the catheter and the finger came in contact. The problem was now solved; there was a communication between the fundus of the bladder and the cervix uteri, about three-quarters of an inch within the cervix, just at its internal orifice, and as large as to admit readily a No. 6 male catheter. After making one of these examinations, a circumstance occurred which confirmed the author in a plan he had devised for the relief of the disease. For the first time for five years she remained dry for two hours; no dribbling took place, and she then passed water in a stream. This arose, he imagines, from the tumefaction of the edges of the fistula producing temporary closure of its orifice. In July, 1841, he acted upon his plan, by proceeding to operate in the following manner:—A Brodie's catheter, armed with a long piece of twine, was introduced into the bladder, concavity downwards; and being guided by a finger within the os uteri, he readily found the rupture, passed the instrument through the vagina, seized the thread, and then withdrew the catheter, thus leaving one end of the thread hanging from the urethra, and the other from the vagina. To the urethral extremity he tied a skein of six threads of glover's silk, oiled them, and drawing at the vaginal extremity, dragged the silk through the fistula, and then tied the urethral and vaginal ends together. The result was most gratifying. From the time of their introduction to the withdrawal of the last thread, no dribbling took place, except when the patient was using some extraordinary bodily exertion; and under these circumstances it did sometimes occur. On the 4th day after the operation, irritation beginning to be produced, the first thread was withdrawn; on the 5th the second; on the 6th the third; on the 10th the fourth; and on the 17th the fifth, leaving only one remaining;—so that in seventeen days the operation may be said to have been completed. During this time considerable irritability of the bladder existed, with great impatience of its contents, so that they were obliged to be evacuated every hour. This symptom, however, gradually subsided simply with fomentations, and without the aid of any medicines. At each recurring menstrual period the urine was, and now is, voided highly charged with the catamenial fluid. During the three subsequent months Mr. Harrinson changed the size of the thread three times, so that at last it was reduced to as small a one as could be procured compatible with any strength and durability. In October the narrator withdrew the last thread. Unfortunately he was disappointed: in twenty-five days the dribbling was as bad as ever. Mr. Harrinson was glad enough, therefore, to retreat to his former position, by introducing now four large threads. In twenty-eight days they were reduced to one, and the patient was herself again. Mr. Harrinson has purposely delayed the publication of the case, in order to test the sufficiency of the means employed. He is pleased to add (May, 1845,) that she remains in the same comfortable state. She changes the thread herself once in three or four weeks, goes about, performs her domestic duties, &c., with no inconvenience. Not a drop of urine has escaped for the last three years. The only precaution necessary is, that the thread be drawn down once a day, to cleanse it from the sabulous matter which is deposited upon it.—*Abridged from the Provincial Med. and Surg. Journal.*

